# MicroRNA-21 inhibits p57^Kip2^ expression in prostate cancer

**DOI:** 10.1186/1476-4598-13-212

**Published:** 2014-09-12

**Authors:** Sweta Mishra, Chun-Lin Lin, Tim H-M Huang, Hakim Bouamar, Lu-Zhe Sun

**Affiliations:** Department of Cellular and Structural Biology, University of Texas Health Science Center, 7703 Floyd Curl Drive, Mail Code 7762, San Antonio, TX 78229-3900 USA; Department of Molecular Medicine, University of Texas Health Science Center, San Antonio, TX USA; Cancer Therapy and Research Center, University of Texas Health Science Center, San Antonio, TX USA

**Keywords:** p57^Kip2^, microRNA-21 and prostate cancer

## Abstract

**Background:**

p57^Kip2^, a cyclin-dependent kinase inhibitor, is considered to be a candidate tumor suppressor gene that has been implicated in Beckwith-Wiedemann syndrome and sporadic cancers. In addition, decreased expression of p57^Kip2^ protein has been frequently observed in pancreatic, lung, breast, bladder, gastrointestinal tract and prostate cancers. However, p57^Kip2^ gene mutations are rare in these cancers suggesting that other unknown mechanisms might be at play in reducing its expression. The aim of this study was to investigate the molecular mechanism of down-regulation of p57^Kip2^ in prostate cancer.

**Findings:**

We observed a significant negative correlation between the expression of p57^Kip2^ and microRNA-21 (miR-21) in prostate cancer samples and after androgen deprivation with castration in the CWR22 human prostate cancer xenograft model. We report that miR-21 targeted the coding region and decreased p57^Kip2^ mRNA and protein levels in prostate cancer cells. Conversely, inhibition of endogenous miR-21 by an anti-miR-21 inhibitor strongly induced p57^Kip2^ expression. Furthermore, we found that knockdown of p57^Kip2^ reversed the effects of the anti-miR-21 inhibitor on cell migration and anchorage-independent cell growth.

**Conclusions:**

Our results indicate that miR-21 is able to downregulate p57^Kip2^ expression by targeting the coding region of the gene and is also able to attenuate p57^Kip2^ mediated functional responses. This is the first report demonstrating that p57^Kip2^ is a novel target of miR-21 in prostate cancer and revealing a novel oncogenic function of this microRNA.

**Electronic supplementary material:**

The online version of this article (doi:10.1186/1476-4598-13-212) contains supplementary material, which is available to authorized users.

## Introduction

Uncontrolled cell proliferation due to aberrant regulation of cell cycle control can lead to the development of cancer. Cyclin dependent kinase inhibitors (CKIs) are the common inhibitors of cell cycle which consists of two families: INK4 family (consisting of p16^INK4a^, p15^INK4b^, p18^INK4c^ and p19^INK4d^) and Cip/Kip family (p21^CIP1/WAF1^, p27^Kip2^ and p57^Kip2^) [1]. Human p57^Kip2^ gene is maternally expressed and paternally imprinted and is located on chromosome 11p15.5 [2], which is implicated in Beckwith-Wiedemann syndrome [3] and in sporadic cancers. p57^Kip2^ is also required for normal development as p57^Kip2^ null mice die at 2 weeks of age and show increased apoptosis and delayed differentiation during mouse development [4]. Because of its chromosomal location, imprinting status and functional activities, p57^Kip2^ is considered to be a candidate tumor suppressor gene. p57^Kip2^ overexpression in LNCaP prostate cancer cells resulted in the conversion of adenocarcinoma to a more differentiated squamous tumor in nude mice, with reduced cell proliferation and tumor invasion [5]. p57^Kip2^ is considered to be a tumor suppressor gene since it functions to block cell proliferation by inhibiting cell cycle progression, promotes apoptosis and cell differentiation, inhibits tissue invasion and metastasis and also inhibits angiogenesis [6]. Hence, cancer cells frequently down-regulate p57^Kip2^ in order to gain a proliferative advantage.

Loss of or reduced p57^Kip2^ expression occurs in carcinomas of the prostate, bladder, liver, pancreas, breast and others. The absence of p57^Kip2^ gene mutations in a wide variety of cancers suggests that other transcriptional or post-transcriptional mechanisms might be involved in its down-regulated protein expression. Inactivation of p57^Kip2^ gene due to promoter DNA methylation was observed in non-small cell lung cancer and in lymphoid malignancies of B-cells [7, 8]. Yang et al. found that p57^Kip2^ expression in breast cancer cells was repressed due to Polycomb protein EZH2-mediated H3K27me3 chromatin mark. Increased p57^Kip2^ degradation due to ubiquitylation by E3 ligase Skp1/ Cul1/ F-box (SCF complex) was observed in non-small cell lung carcinoma and hepatocellular carcinoma [9, 10].

Mature miRNAs are ~22 nucleotides long non-coding single-stranded RNAs, which upon binding to the 3’-UTR region of target mRNAs can result in mRNA cleavage, or translational repression. p57^Kip2^ has been reported to be targeted by miR-221/222 cluster in gastric carcinoma, ovarian cancer and hepatocellular carcinoma [11]. miR-92b and miR-25 have also been reported to down-regulate p57^Kip2^ expression in human embryonic stem cells and in gastric cancer respectively [12, 13]. Jin et al. demonstrated that expression of p57^Kip2^ is significantly decreased in human prostate cancer and overexpression of p57^Kip2^ in prostate cancer cells decreased cell proliferation and reduced invasiveness [5]. However, the mechanism behind p57^Kip2^ down-regulation in prostate cancer cells has not been investigated. Unexpectedly, we discovered that p57^Kip2^ is one of the novel downstream target genes of miR-21 in prostate cancer. We observed a very strong negative correlation between p57^Kip2^ and miR-21 expression in human prostate tumor samples and in CWR22, a human prostate cancer xenograft model. Our results for the first time show that miR-21 can down-regulate p57^Kip2^ mRNA and protein expression by targeting its coding region to attenuate its activity in prostate cancer cells. Thus, therapeutic approaches aimed at restoring p57^Kip2^ expression might be beneficial for prostate cancer prevention and therapy.

## Findings and discussion

### MicroRNA-21 targets p57^Kip2^ gene

**Figure 1:**
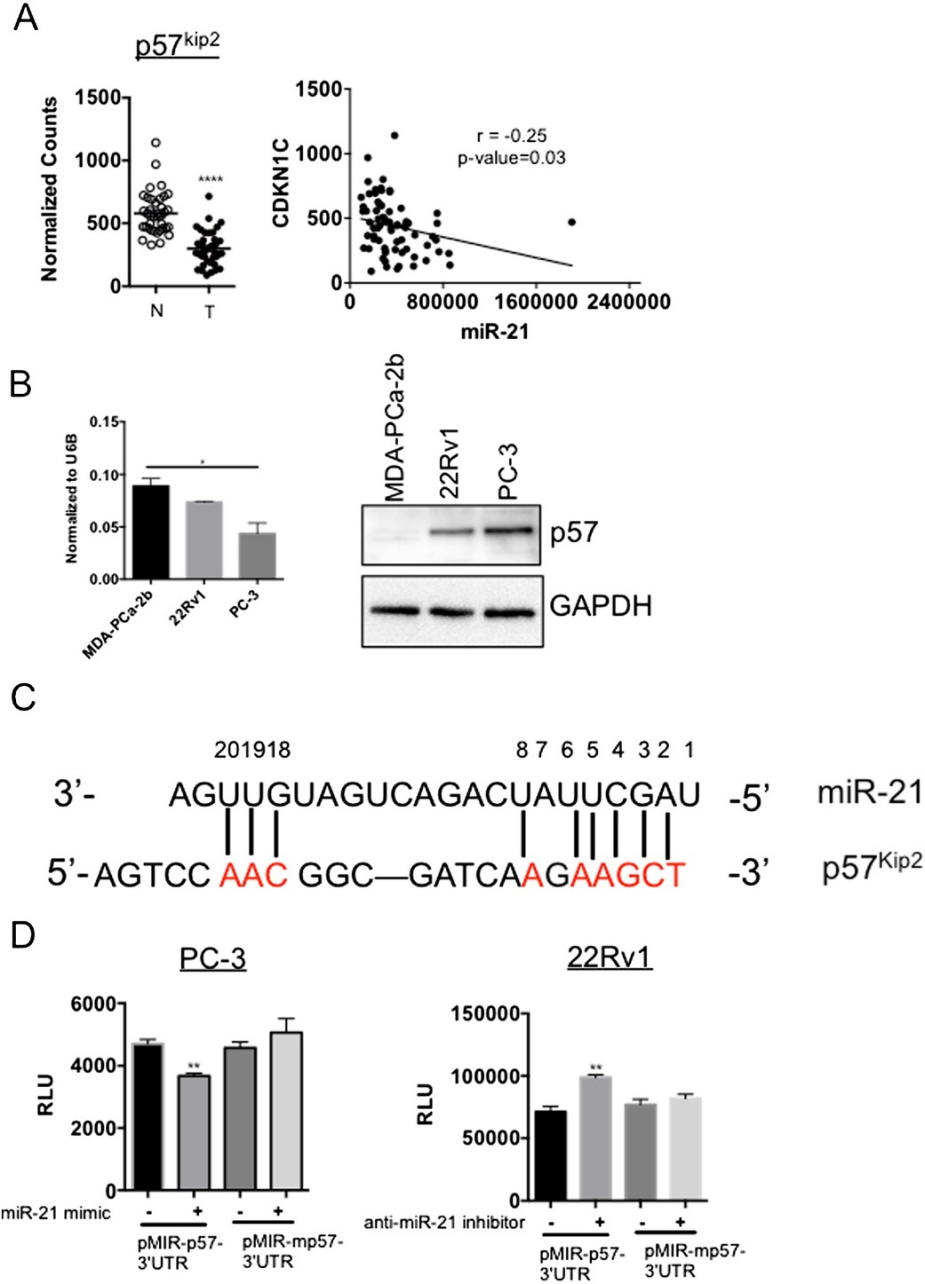
**MicroRNA-21 targets p57** ^**Kip2**^ **gene. (A)** miR-21 and p57^Kip2^ are inversely correlated in 37 matched normal (N) and tumor (T) samples downloaded from the TCGA database. r: Pearson product-moment correlation coefficient. ****P < 0.0001 with one-way analysis of variance. **(B)** miR-21 (left panel) and p57^Kip2^ (right panel) levels were measured in prostate cancer cells with real-time RT-PCR and western blotting respectively. *P < 0.05 with one-way analysis of variance test. **(C)** Sequence alignment between miR-21 seed sequence and a partial coding or mutated sequence of p57^Kip2^. **(D)** Luciferase assay was performed with miR-21 mimic or anti-miR-21 transfection in cells with co-transfection of pMIR-3’UTR- p57^Kip2^ or pMIR-3’UTR-mutated p57^Kip2^ and a ß-Gal expression construct. **P < 0.01 with one-way analysis of variance and Tukey-Kramer *post hoc* test.

Inactivation of p57^Kip2^ is commonly observed in cancers. MicroRNAs offer another layer of complexity to the regulation of its gene expression. Recently, microRNAs (miR-221/222, miR-25, miR-92b) were reported to downregulate the expression of p57^Kip2^ transcript [13, 14]. In our studies, we found a very significant p57^Kip2^ downregulation in human prostate tumor samples compared to the normal prostate cells in consistence with the published studies [5, 15]. However, the mechanism of p57^Kip2^ repression has not been well studied in prostate cancer. We along with others have shown a significant upregulation in miR-21 expression in prostate cancer [16, 17]. Surprisingly, we found a significant (P < 0.05) inverse correlation between miR-21 and p57^Kip2^ expression in human prostate cancer samples downloaded from the Cancer Genome Atlas (TCGA) database (Figure 1A). We also checked miR-21 expression in different prostate cancer cells and found that all the cells expressed miR-21 albeit at different levels. Cells with relatively higher miR-21 levels such as MDA-PCa-2b and 22Rv1 expressed lower levels of p57^Kip2^ when compared to PC-3 cells that expressed relatively low miR-21 and higher p57^Kip2^ (Figure 1B). Hence, we hypothesized that p57^Kip2^ may be a downstream target gene of miR-21 in prostate cancer. Regulation of target genes by binding of microRNA’s to the 3' untranslated regions (UTRs) of their mRNA’s has been well characterized. Hence, most of the bioinformatics tools available to predict miRNA target sites on mRNAs have been mainly focused on target sites within the 3'-UTRs of genes. A study by Hausser et al. suggest that miRNAs may combine targeting of coding regions and 3’ UTRs to flexibly tune their post-transcriptional regulatory effects [18]. Available microRNA prediction target algorithms did not predict p57^Kip2^ gene to be targeted by miR-21. However, scanning of human p57^Kip2^ gene for the miR-21 recognition site, revealed a 5-mer complementary binding site to the 2-7 seed region of mature miR-21 and a 3-mer sequence complementary to the 3’-target site (18-20 nucleotide) in the coding region of p57^Kip2^, which is from nucleotides 1054 to 1077 in accession # NM_00007 of GenBank as shown in Figure 1C. To experimentally test if miR-21 targets p57^Kip2^, we cloned this putative miR-21 recognition sequence of p57^Kip2^ in the 3’-UTR region of the luciferase gene in the pMIR reporter vector. Transfection of miR-21 mimic in PC-3 prostate cancer cells significantly (P < 0.01) reduced the luciferase activity (Figure 1D). However, mutation of the putative miR-21 binding nucleotides (Figure 1C and Additional file 1) abolished this effect. Similarly, luciferase activity was significantly (P < 0.01) increased when 22Rv1 prostate cancer cells, which were previously also shown to express relatively high endogenous miR-21 [16], were transfected with an anti-miR-21 to inhibit the endogenous miR-21 levels (Figure 1D), and the mutation of the miR-21 binding sites abrogated this effect.

### MicroRNA-21 downregulates p57^Kip2^ expression

**Figure 2:**
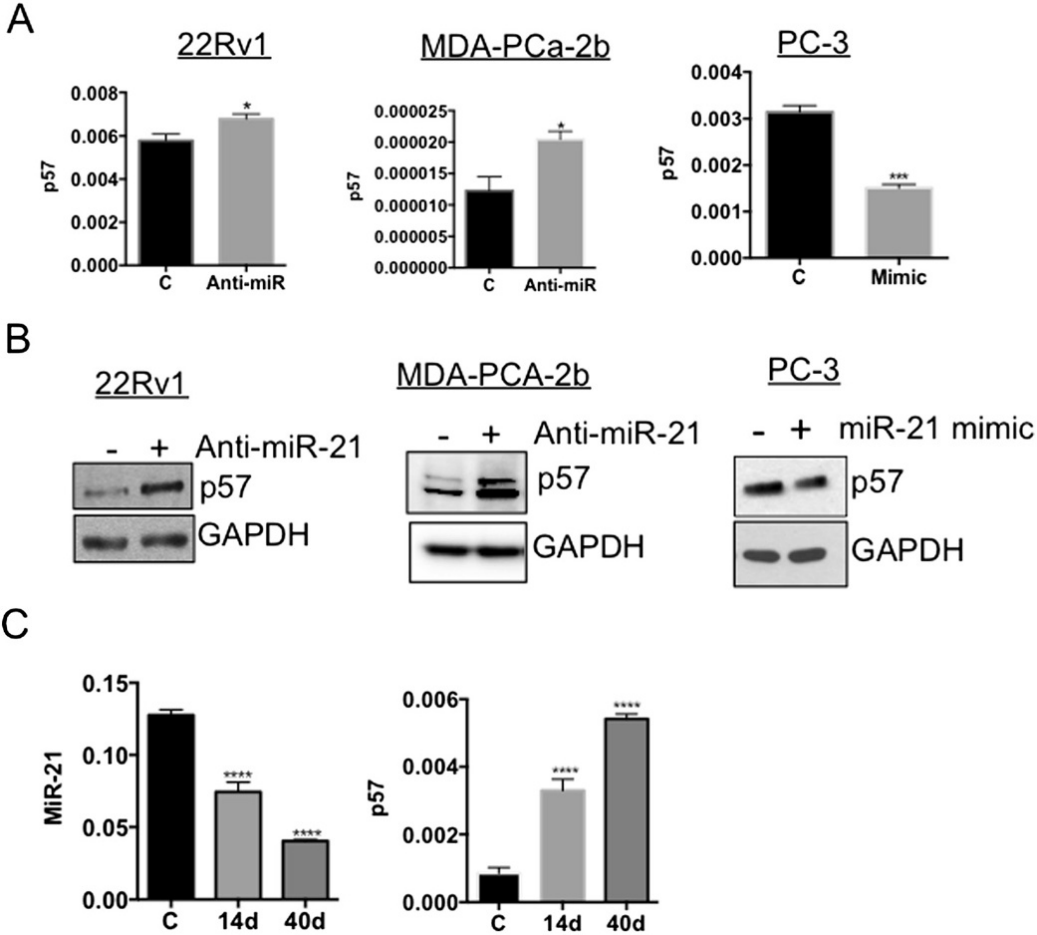
**MicroRNA-21 downregulates p57** ^**Kip2**^ **in prostate cancer cells. (A)** 22Rv1 and MDA-PCa-2b cells were transfected with the inhibitor negative control and anti-miR-21 (50 nM) for 48 h. PC-3 cells were transfected with control siRNA and miR-21 mimic (20 nM) for 48 h. p57^Kip2^ gene expression was checked by real-time RT-PCR. *P < 0.05 and ***P < 0.001 with a Student’s *t*-test analysis. **(B)** Cell lysates were used for Western analysis to measure the level of p57^Kip2^ and GAPDH after anti-miR-21 inhibitor or miR-21 mimic transfection for 48 h. **(C)** p57^Kip2^ and miR-21 expressions were analyzed in CWR22 tumors after 14 and 40 days of castration. ****P < 0.0001 with one-way analysis of variance test.

To further validate these findings, we checked the effect of miR-21 on p57^Kip2^ mRNA and protein levels. Inhibition of endogenous miR-21 with an anti-miR-21 in 22Rv1 and MDA-PCa-2b cells increased the p57^Kip2^ mRNA levels (P < 0.05), whereas miR-21 mimic decreased (P < 0.001) the basal level of p57^Kip2^ in PC-3 cells (Figure 2A). Transfection of anti-miR-21 in 22Rv1 and MDA-PCa-2b cells increased the p57^Kip2^ protein expression and miR-21 mimic transfection in PC-3 decreased p57^Kip2^ expression (Figure 2B). We used a CWR22 xenograft mouse model to study the effect on microRNA-21 and p57^Kip2^ expression under androgen depletion with castration [19]. We have previously shown that castration led to down-regulation of miR-21 as androgen signaling was shown to stimulate miR-21 [16]. We harvested tumors for miR-21 and p57^Kip2^ expression analysis at 14 and 40 days post castration, respectively. The results showed a significant (P < 0.0001) decrease in miR-21 expression levels in the castrated mice when compared to control non-castrated mice (Figure 2C) as was observed in our previous study. Interestingly, we observed an increase in p57^Kip2^ expression at 14 and 40 days in the castrated mice showing an inverse correlation between miR-21 and p57^Kip2^ expression in vivo (Figure 2C) (P < 0.0001). We show that for the first time, castration-mediated miR-21 decreases correlate with an increase in p57^Kip2^ under in vivo conditions. Currently, it is not clear how androgen/AR signaling regulates p57^Kip2^ expression in prostate cancer cells. Our results suggest that androgen/AR signaling-mediated inhibition of p57^Kip2^ expression appears to be mediated by miR-21 in CWR22 human prostate cancer xenografts. Further studies are needed to understand whether p57^Kip2^ expression contributes to castration-induced regression of prostate cancer.

### MicroRNA-21 abrogates p57^Kip2^-mediated functional responses in prostate cancer cells

**Figure 3:**
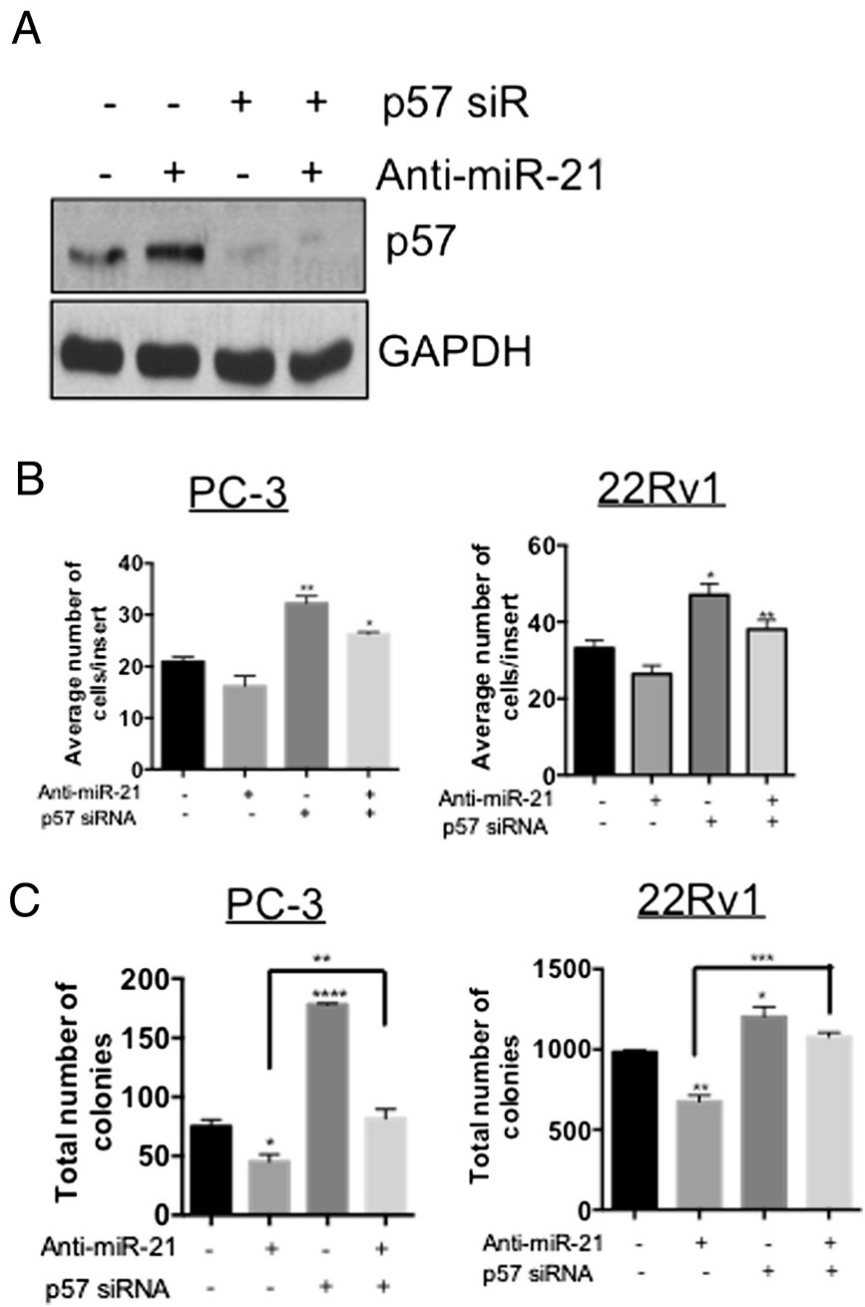
**MicroRNA-21 attenuates p57** ^**Kip2**^ **mediated functional responses in prostate cancer cells.** Cells were transfected with 50 nM of control siRNA, anti-miR-21 or p57^Kip2^ siRNA either alone or in combination for 48 h. Cells were trypsinized and replated for Western analysis, cell migration or soft agar assay. **(A)** Western analysis in PC-3 cells for p57^Kip2^ and GAPDH was performed. **(B)** Number of cells migrated were quantified. *P < 0.05 and **P < 0.01 with one-way analysis of variance test. **(C)** After 14 days, colonies were stained and quantified in PC-3 and 22Rv1 cells for soft-agar colony formation assay. *P < 0.05, **P < 0.01 and ***P < 0.001 with one-way analysis of variance test.

p57^Kip2^ has been shown previously to act as a tumor suppressor gene by inhibiting cell migration and invasion [20]. Hence, we tested whether endogenous miR-21 functionally abrogated p57^Kip2^ mediated tumor suppressive responses in prostate cancer cells. When we inhibited the endogenous miR-21 in PC-3 cells with an anti-miR-21 inhibitor, we observed an induction in p57^Kip2^ expression (Figure 3A). However, co-transfection with a p57^Kip2^ small-interfering RNA with anti-miR-21 inhibitor abolished the p57^Kip2^ induction by anti-miR-21 (Figure 3A). We also found that p57^Kip2^ inhibition significantly increased the cell migration in both PC-3 and 22Rv1 cells (Figure 3B). Transfection of cells with an anti-miR-21 inhibitor decreased the cell migration, although the results were not statistically significant. Interestingly, we found that the co-transfection of cells with anti-miR-21 and p57^Kip2^ siRNA, significantly increased cell migration when compared to anti-miR-21 treatment alone (Figure 3B) (P < 0.05 and P < 0.01). Similarly, while knocking down of endogenous miR-21 significantly reduced anchorage-independent growth, knocking down p57^Kip2^ reversed the inhibitory effect of the anti-miR-21 inhibitor on the anchorage-independent cell growth (Figure 3C) (P < 0.01 and P < 0.001). These results show that the functional effects of miR-21 in prostate cancer cells could be mediated partly by a downregulation in p57^Kip2^ expression.

In summary, we discovered p57^Kip2^ to be a novel target gene of microRNA-21 in prostate cancer. Our findings provide a novel mechanism of p57^Kip2^ downregulation in prostate cancer. These findings warrant further research to test if p57^Kip2^ is also a novel target gene of miR-21 in other cancer types as well. In our previous study, we found miR-21 to be an oncogenic regulator in prostate cancer by targeting the tumor suppressive effects of TGF-beta signaling pathway in cancer cells [16]. Given, the regulation of p57^Kip2^ by microRNA-21 in the current study, we provide a strong rationale to perform preclinical testing of microRNA-21 inhibitor as a novel therapeutic drug for prostate cancer.

## Electronic supplementary material

Additional file 1: **Materials and methods.** (DOCX 30 KB)
