# Supplementary material for: MicroRNA-21 inhibits p57Kip2 expression in prostate cancer
Source: Mol Cancer. 2014 Sep 12;13:212. doi: 10.1186/1476-4598-13-212 (PMC4168249; doi:10.1186/1476-4598-13-212)
Supplement: Supplementary file 1 — Additional file 1: Materials and methods. (DOCX 30 KB) [file 12943_2014_1409_MOESM1_ESM.docx]

**Additional file 1. Materials and Methods**

**Cell Culture.** 22Rv1, MDA-PCa-2b and PC-3 cells were purchased from the American Type Culture Collection (ATCC, Manassas, VA, USA). The cells were cultured in medium recommended by ATCC and maintained at 37ºC in a 5% carbon dioxide humidified incubator.

**Cloning, transient transfection and in vitro luciferase assay.** Forward and reverse primers containing SpeI and HindIII overhangs were designed using human p57^Kip2^ mRNA as template with putative microRNA-21 recognition site in the coding region. pMIR luciferase construct was used to clone the microRNA-21 recognition sites in the 3’-UTR region of luciferase gene to test whether miRNA-21 targets this recognition sequence. The primers were annealed and ligated to the SpeI-Hind III digested pMIR vector construct (Ambion, Austin, Tx, USA). The oligonucleotides used in our study were pMIR-p57^Kip2^, 5’- CTAGTCCAACGGCGCGGCGATCAAGAAGCTGA-3’ and 5’-AGCTTCAGCTTCTTGATCGCCGCGCCGTTGGA-3’, pMIR-mutated p57^Kip2^, 5’-CTAGTCCTAGGGCGCGGCGATCAAGTACGAGA-3’ and 5’-AGCTTCTCGTACTTGATCGCCGCGCCCTAGGA-3’. For transient transfection, a total of 0.5 x 10^5^ cells were plated in 24-well plate, or 0.1 x 10^6^ cells in 12-well plate 12 hours prior to transfection. Plasmid DNA (100 ng) was co-transfected with 50 ng of ß-Gal and miR-21 mimic or anti-miR-21 with lipofectamine 2000 (Invitrogen, Carlsbad, CA, USA) transfection reagent. All Stars negative control small interfering RNA (Qiagen, Valencia, CA, USA) and miScript Inhibitor Negative Control (Qiagen) were used as controls for mimic or inhibitor transfection respectively. After 24 h of incubation, luciferase assay was performed as described previously.

**The Cancer Genome Atlas Database (TCGA) analysis.** Microarray data from TCGA database was downloaded for samples and regression analysis was plotted using GraphPad Prism 6 software (GraphPad Prism, La Jolla, CA, USA).

**Western Blot Analysis.** Samples after harvesting were lysed in Laemmli buffer containing protease inhibitors. BCA protein assay (Thermo Scientific, Rockford, IL) was used to quantify total protein concentrations. An equal amount of protein was resolved by SDS-PAGE, transferred to a nitrocellulose membrane under constant voltage and blocked with 5% non-fat dried milk in TBST (10mM Tris pH 7.5, 150mM NaCl and 0.05% Tween-20) followed by TBST washings and incubation in primary antibodies overnight. Secondary antibodies were applied next day with three TBST washes in between. Proteins were detected using the Amersham ECL western blotting detection kit (GE Healthcare, Piscataway, NJ). Primary antibody to p57^Kip2^ was purchased from abcam (Cambridge, MA, USA) and GAPDH from CalBioChem (Billeria, MA, USA).

**RNA extraction and quantitative reverse transcriptase PCR (qRT-PCR) analysis.** Cells were lysed with TRIzol reagent (Sigma, St Louis, MO, USA) for dissociation of any RNA-protein complexes. Chloroform was added for phase separation, followed by the precipitation of aqueous phase containing total RNA using isopropyl alcohol. The RNA pellet after centrifugation was washed in 75% ethanol and suspended in nuclease-free water. The concentration and quality of RNA was estimated using Nanodrop analysis with an absorbance at 260nm for RNA measurement and a 280nm absorbance for protein concentration. 2ug of total RNA was reverse transcribed to make cDNA using High Capacity cDNA Reverse Transcription kit (Applied Biosystems, Foster City, CA, USA) with random primers. After reverse transcription, quantitative real time- PCR was performed with the gene specific forward and reverse primers after diluting the cDNA (1:5) using SYBR Green dye (ABI, Foster City, CA, USA). Human p57^Kip2^ was amplified using forward 5’-CAACCACCAGGGCATCCA-3’ and reverse 5’-TCGTGGTCCCAGCACTCA’3’, Real-time PCRs were performed using Step One Plus Real Time PCR System (Applied Biosystems). Total RNA containing miRNA was isolated using miRNeasy kit (Qiagen) according to the manufacturer’s protocol. β-Actin and U6B was used as an endogenous control for mRNA and miRNA measurements. Result was expressed as 2(^-∆Ct^) method.

**Cell Migration Assay.** Migration assays were performed using 24-well Boyden chambers using 8um polycarbonate membrane pores (BD Biosciences, Durham, NC, USA). Cells after transfection for 48 hours were seeded in the upper chamber at 50,000 cells/ insert for PC-3 and 80,000 cells/insert for 22Rv1 cells in serum-free medium in triplicates. Complete medium was added to the bottom well that acted as the chemo attractant. After 18h of incubation in 37ºC with 5% carbon dioxide humidified incubator, cells were stained with Hema 3 Stain 18 kit (Fischer Scientific, Waltham, MA, USA) according to the manufacturer’s protocol. Stained cells were counted under the microscope with 100X magnification and quantified.

**Animal Experiments.** Four- to five-week-old male athymic nude mice were purchased from Harlan Sprague-Dawley, Inc. Animals were maintained under the care and supervision of the Laboratory Animal Research facility at the University of Texas Health Science Center, San Antonio, Texas. The animal protocol was approved and monitored by the Institutional Animal Care and Use Committee. CWR22 human xenograft tumors were obtained from Clifford G. Tepper at UC Davis Cancer Center and maintained by serial passaging in male nude mice injected with testosterone pellet. 0.07g of tumor cell suspension was mixed with an equal volume of matrigel, and was subcutaneously implanted on the lower back of male nude mice. After four weeks, testosterone pellet was removed and mice were surgically castrated. Control mice were left on a 90day slow release testosterone pellet. After 14 and 40 days of castration, tumors from two mice in each group were harvested for RNA isolation using RNeasy Plus kit (Qiagen, Valencia, CA). The flow through was used for microRNA isolation using miRNeasy kit (Qiagen). Three control mice were also sacrificed for tumor isolation.

**Soft Agar Colony Formation Assay.** Cells after transfection for 48 hours were trypsinized and plated at 3000 cells/well for 22Rv1 and 2000 cells/ well for PC-3 in 500 uL of 0.4% low melting agarose (Invitrogen) on top of an existing 0.8% agarose layer in a 12-well plate in triplicates. The plate was incubated in a 5% carbon dioxide humidified incubator for two weeks. Colonies were counted after staining with p-iodonitrotetrazolium violet (Sigma) for 24 hours.

**Statistical Analysis.** Two-tailed student’s t-test was used to compare two groups. One-way analysis of variance was used to analyze data with more than two groups using Tukey-Kramer *post-hoc* test. Results are expressed as mean ± sem. P<0.05 was considered as statistically significant. The statistical analysis was performed using GraphPad Prism 6 software.
